# Inhibition of γ-glutamyl transferase suppresses airway hyperresponsiveness and airway inflammation in a mouse model of steroid resistant asthma exacerbation

**DOI:** 10.3389/fimmu.2023.1132939

**Published:** 2023-06-12

**Authors:** Cancan Zhang, Huisha Xu, Keilah G. Netto, Leon A. Sokulsky, Yiyan Miao, Zhongyuan Mo, Yan Meng, Yingying Du, Chengyong Wu, Liyou Han, Lirong Zhang, Chi Liu, Guojun Zhang, Fuguang Li, Ming Yang

**Affiliations:** ^1^ Academy of Medical Sciences & Department of Immunology, School of Basic Medical Sciences, Zhengzhou University, Zhengzhou, China; ^2^ Priority Research Centre for Healthy Lungs, School of Biomedical Sciences & Pharmacy, Faculty of Health and Hunter Medical Research Institute, The University of Newcastle, Callaghan, New South Wales, Australia; ^3^ Institute for Liberal Arts and Sciences, Kyoto University, Kyoto, Japan; ^4^ Department of Pharmacology, School of Basic Medical Sciences, Zhengzhou University, Zhengzhou, China; ^5^ Department of Physiology, School of Basic Medicine Science, Central South University, Changsha, China; ^6^ Department of Pulmonary and Critical Care Medicine, The First Affiliated Hospital of Zhengzhou University, Zhengzhou, China

**Keywords:** asthma exacerbation, LPS, γ-glutamyl transferase, airway hyperresponsiveness, airway inflammation

## Abstract

**Introduction:**

Despite recent advances, there are limited treatments available for acute asthma exacerbations. Here, we investigated the therapeutic potential of GGsTop, a γ-glutamyl transferase inhibitor, on the disease with a murine model of asthma exacerbation.

**Methods:**

GGsTop was administered to mice that received lipopolysaccharide (LPS) and ovalbumin (OVA) challenges. Airway hyperresponsiveness (AHR), lung histology, mucus hypersecretion, and collagen deposition were analyzed to evaluate the hallmark features of asthma exacerbation. The level of proinflammatory cytokines and glutathione were determined with/without GGsTop. The transcription profiles were also examined.

**Results:**

GGsTop attenuates hallmark features of the disease with a murine model of LPS and OVA driven asthma exacerbation. Airway hyperresponsiveness (AHR), mucus hypersecretion, collagen deposition, and expression of inflammatory cytokines were dramatically inhibited by GGsTop treatment. Additionally, GGsTop restored the level of glutathione. Using RNA-sequencing and pathway analysis, we demonstrated that the activation of LPS/NFκB signaling pathway in airway was downregulated by GGsTop. Interestingly, further analysis revealed that GGsTop significantly inhibited not only IFNγ responses but also the expression of glucocorticoid-associated molecules, implicating that GGsTop profoundly attenuates inflammatory pathways.

**Conclusions:**

Our study suggests that GGsTop is a viable treatment for asthma exacerbation by broadly inhibiting the activation of multiple inflammatory pathways.

## Introduction

Hospitalizations as a result of asthma exacerbations are common, especially in children, and account for a significant proportion of lung associated healthcare costs (1, 2). Environmental agents including air pollutants and airway infection are the major trigger of exacerbations. Asthma exacerbations are associated with a decline in lung function and heightened inflammation of the airways (2). Both animal and clinical studies have demonstrated that the inflammatory response of asthma exacerbations is featured by the infiltration of a mixture of multiple immune cells including eosinophils and neutrophils, and is strongly linked to many proinflammatory cytokines and chemokines including type 1 and 2 cytokines, tumor necrosis factor (TNF)-α, MCP-1 and IL-6 (3, 4). These observations are indicative that the complex and multifactorial pathophysiological changes in the airways are characteristic with the pathogenesis of asthma exacerbations.

Asthma exacerbations are often difficult to control with conventional glucocorticosteroids-based therapies and novel approaches to therapy are urgently needed (1, 5, 6). Currently, there has been an influx of biologic treatments for asthma targeting cytokines that are notable drivers for asthma exacerbation, such as tezepelumab (anti-TSLP), astegolimab (anti-ST2), itepekimab (anti-IL-33), and risankezumab (anti-IL-23) (7–10). While there has been some success in employing these biologic agents as stand-alone therapies or add-ons to glucocorticoids, issues regarding the high cost of these therapeutics remain to be a barrier to access (11). Furthermore, most biologic therapies are tailored towards type 2 immune factors (namely TSLP, IL-5, and IL-4R), and biologics developed for non-Th2 driven exacerbations (e.g. anti-IL-17) have not demonstrated adequate efficacy (12).

γ-glutamyl transferase (GGTs) are ubiquitous enzymes that hydrolyze glutathione S-conjugates and γ-glutamyl bonds of glutathione (GSH) (13). This results in the degradation of extracellular GSH, which is a crucial process involved in the equilibrium of intracellular and extracellular redox reactions (14). Increased expression of GGT in the bloodstream is an indicator of liver or bile duct damage. Accumulating evidence shows that GGTs are crucial to the pathogenesis of many human diseases through oxidative stress dysregulation, including ischemia, cancer, and lung inflammation (15–17). Interestingly, GGsTop, an effective and non-toxic inhibitor of GGTs (18), has been shown to improve outcomes in GGTs-related diseases including cancer, lung inflammation, neuroinflammation, and ischemia-reperfusion (19–24). Additionally, GGsTop was also successful in attenuating IL-13-induced airway hyperreactivity and mucous production (20), suggesting that there is therapeutic potential for this compound in the treatment of asthma. However, studies with GGT-deficient mice and GGsTop administration have employed IL-13 to induce airway pathological response, which is specifically focused on type 2-associated pathways, consisting of eotaxins induced eosinophilic, MUC5AC-muscus hypersecretion, and IL-13 induced AHR (25, 26). By contrast, OVA/LPS-based model has a complex immune response in the lung that not only enhances type 2 response including IL-13 but also involves type 1 response including IFNγ, and aberrant macrophage activation (27–29), which better reflects the pathological changes in the lung during asthma exacerbation. Moreover, the effectiveness of GGsTop in OVA/LPS-induced asthma exacerbation has never been investigated.

Here, we examined the effect of GGsTop in our established mouse model of lipopolysaccharide (LPS) and ovalbumin (OVA) induced steroid-resistant asthma, by assessing the therapeutic outcomes of airway hyperresponsiveness (AHR), airway inflammation and mucus secretion. The reason of employing this model is that there is due to the association between steroid-resistant exacerbations and the level of LPS exposure epidemiologically (30, 31). We further investigated how GGsTop affected the downstream inflammatory pathways and outlined the role of GGTs in asthma exacerbation. Overall, our results suggest that GGsTop has therapeutic potential for patients with steroid resistant asthma exacerbations, and encourages further investigation into GGTs and GSH in the propagation of asthmatic phenotypes.

## Materials and methods

### Mice

Specific pathogen-free BALB/c male mice (6–8 wk old) were obtained from Beijing Vitonlihua Company [license No. SCXK (Beijing) 2021-0006] (Beijing, China) and were housed in an SPF environment at the animal housing facility, Zhengzhou University. All experimental protocols were approved by the Animal Ethics Committee of Zhengzhou University (#ZZURIB20180120).

### Establishment of asthma exacerbations

Mice were sensitized by intraperitoneal (i.p) injection consisting of a mixture of OVA and Alum hydroxide gel as previously described (32). The ratios consisted of 50µg of OVA and 2mg Alum (Sigma‐Aldrich) in 200 µL sterile PBS per mouse on days 0 and 7. Mice were then challenged by OVA intranasally (i.n) (5 µg in 50 μL sterile PBS) on days 14-17. Nonsensitized mice received sterile PBS only. Where indicated, mice received i.n. LPS (50 ng; Sigma-Aldrich) in PBS on days 19 and 21. Endpoints were assessed on day 25. To inhibit the activity of GGTs, some mice were intratracheally (i.t.) administered GGsTop (0.5mg/kg, WakoPure, Osaka, Japan) or vehicle control on days 21, 22, and 23, according to previously published method (20). GGT activity can be effectively inhibited in BALF and serum after trachea-delivery of this dose.

### Lung function

Airway resistance (Raw) in response to increasing doses of aerosolized methacholine (MCh, Sigma‐Aldrich) was measured using a Flexivent apparatus (FX1 system; Scireq) as previously described (32, 33). Briefly, mice were anesthetized (i.p.) using a combination of xylazine (2 mg/mL, Troy Laboratories), ketamine (40 mg/mL, Parnell), and PBS (ratio of 5:4:1). A cannula was inserted into the airways, and mice were ventilated with a tidal volume of 8 mL/kg at a frequency of 450 breaths/minute. Mice were challenged with saline aerosol followed by increasing concentrations of methacholine (0.1, 0.3, 1, 3, 10, and 30 mg/mL). Measurements were excluded if the coefficient of determination was lower than 95%. Airway resistance was recorded and presented as a percentage increase over baseline.

### Bronchoalveolar lavage fluid cells and lung histology

BALF was collected immediately following lung function measurements. The left lung lobe was tied, and the right lung was flushed three times with 500µl sterile PBS. BALF was centrifuged to pellet cells and red blood cells were then removed by using red blood cell (RBC) lysis buffer. Total cell numbers were determined for each sample before being spun on a microscope slide using a cytospin centrifuge. Cells were then stained with May-Grunwald and Giemsa staining reagents and counted under a 100× microscope to determine percentage populations of immune cells (approximately 500 cells per sample).

Left lung lobes from treated mice were removed, fixed in 10% buffered formalin, embedded, and sectioned (4–6 mm). Sections were then stained with hematoxylin-eosin (HE), periodic acid–Schiff (PAS) or Masson’s Trichrome Stain (MTS). Scorings for histopathology (inflammatory infiltrates), PAS (mucus-producing cells) or MTS (collagen deposition) were performed according to a set of morphological criteria, as previously described (34, 35). Briefly, scoring of airway and vascular Inflammation: 0 = no inflammation, 1 = Some airways/vessels have small numbers of cells, 2 = Some airways/vessels have significant inflammation, 3 = Majority of airways/vessels have some inflammation, 4 = Majority of airways/vessels are significantly inflamed. Scoring of parenchymal Inflammation: 0 = <1% affected, 1 = 1-9% affected, 2 = 10-29% affected, 3 = 30-49% affected, 4 = 50-69% affected, 5 = >70% affected. Airways, bold vessels and parenchyma were scored separately and summed to the total score. Scoring was performed blinded.

### Blunt dissection of airway

Airway tissue was isolated by blunt dissection, using two pairs of forceps to separate lung parenchyma from the larger airways and leaving several generations of airway attached to the trachea (36). Airway tissue without trachea was then snap frozen in liquid nitrogen until RNA extraction was performed.

### Peripheral blood

Peripheral blood was collected from the tail veins of mice by cutting the tail, discarding the first drop of blood, then adding the next drop of blood to one end of the slide. Blood was smeared on the slide and Wright-Giemsa staining was then performed on the slide. The blood smear was dried and sealed, and cells were enumerated using light microscopy as previously described (37).

### Bone marrow

Femurs were isolated using scissors and forceps, taking care that the bone is not broken. The ends of epiphyses were cut off with scissors and RPMI1640 (4 ml/mice) was flushed into the bone marrow cavity with a 1 ml syringe to collect bone marrow cells as previously described. After centrifugation, red blood cells were removed using RBC-lysis buffer. Cells were washed with 5 ml 2%FCS/PBS twice. Total cell numbers were determined for each sample before being spun on a microscope slide using a cytospin centrifuge (approximately 2×10^5^ cells/sample). Cells were then stained with May-Grunwald and Giemsa staining reagents and counted under a 100× microscope to determine percentage populations of immune cells (500 cells per sample) as previously described (37).

### RNA extraction, RT-PCR and quantitative PCR

Total RNA was isolated from bluntly dissected airway tissues using TRIzol reagent (Takara, Beijing, China) and phenol-chloroform extraction and quantified on a Nanodrop 1000 spectrophotometer (ND‐1000; Thermo Scientific). cDNA was synthesized using random hexamer primers and MMLV reverse transcriptase (Invitrogen) on a T100 thermal cycler (Bio‐Rad). Quantitative PCR was performed on a Viia7 real‐time PCR machine (Life Technologies) using SYBR reagents. Thermal cycling conditions consisted of an initial denaturing step (95°C, 3 minutes) followed by 40 cycles of denaturing (95°C, 5 seconds) and annealing (60°C, 30 seconds). Resulting mRNA levels were normalized to GAPDH and expressed as a fold-change relative to control samples (PBS group) (Primers shown in **Supplementary Table 1**).

### Protein measurement

Total lung tissue protein was extracted using RIPA tissue protein lysate (Solarbio, Beijing, China). Total protein concentration was determined using a BCA Protein Assay kit (BestBio, Beijing, China). The levels of IL‐5, IL‐13 and IFNγ were measured using Cytometric Bead Array (CBA) flex set (BD Biosciences, San Jose, CA) according to manufacturer’s instructions. Data were acquired by FACS Cytometry and analyzed with FCAP Array v3 (BD Bioscience). Individual cytokine levels were normalized to total protein.

### Detection of GSH content and GGT enzyme activity

The GSH and GSSG assay kits (Beyotime, Shanghai, China) were used to calculate the GSH content by measuring the absorbance of the products at 412 nm using DTNB as the substrate according to the manufacturer’s instructions. GGT enzyme activity were measured using γ-glutamyl Transpeptidase Activity Assay Kit (Solarbio, Beijing, China). Briefly, GGT catalyzes the transfer of γ-glutamyl from glutamyl-p-nitroanilide to N-glycylglycine to produce p-nitroanilide, which has a characteristic light absorption value at 405 nm. Glutamyl-p-nitroanilide and N-glycylglycine was used as substrates to calculate the activity of GGT enzyme by measuring the rate of increase of light absorption at 405 nm at room temperature. Resulting data were calibrated compared to control groups (PBS group).

### RNA sequencing and bioinformatic analysis

RNA-seq was performed by PANOMIX Biomedical Tech Co., LTD (Suzhou, China). RNAseq data can be accessed in the Gene Expression Omnibus under accession number GSE217834. Differential analysis of gene expression was performed with the R package of DESeq. Gene ontology (GO), KEGG, and Gene set enrichment analysis (GSEA) were performed with the R package of ClusterProfiler.

### Statistical analysis

Statistical analysis was performed using Prism version 6.07 (GraphPad Software). Two‐way ANOVA was used to identify differences between two or more experimental groups, and Student’s unpaired tests were used where comparison was made between two treatment groups. All results were presented as means ± SEM, and P values < 0.05 were considered statistically significant.

## Results

### GGsTop attenuates hallmark features of asthma in a LPS-induced exacerbation model

**Figure 1:**
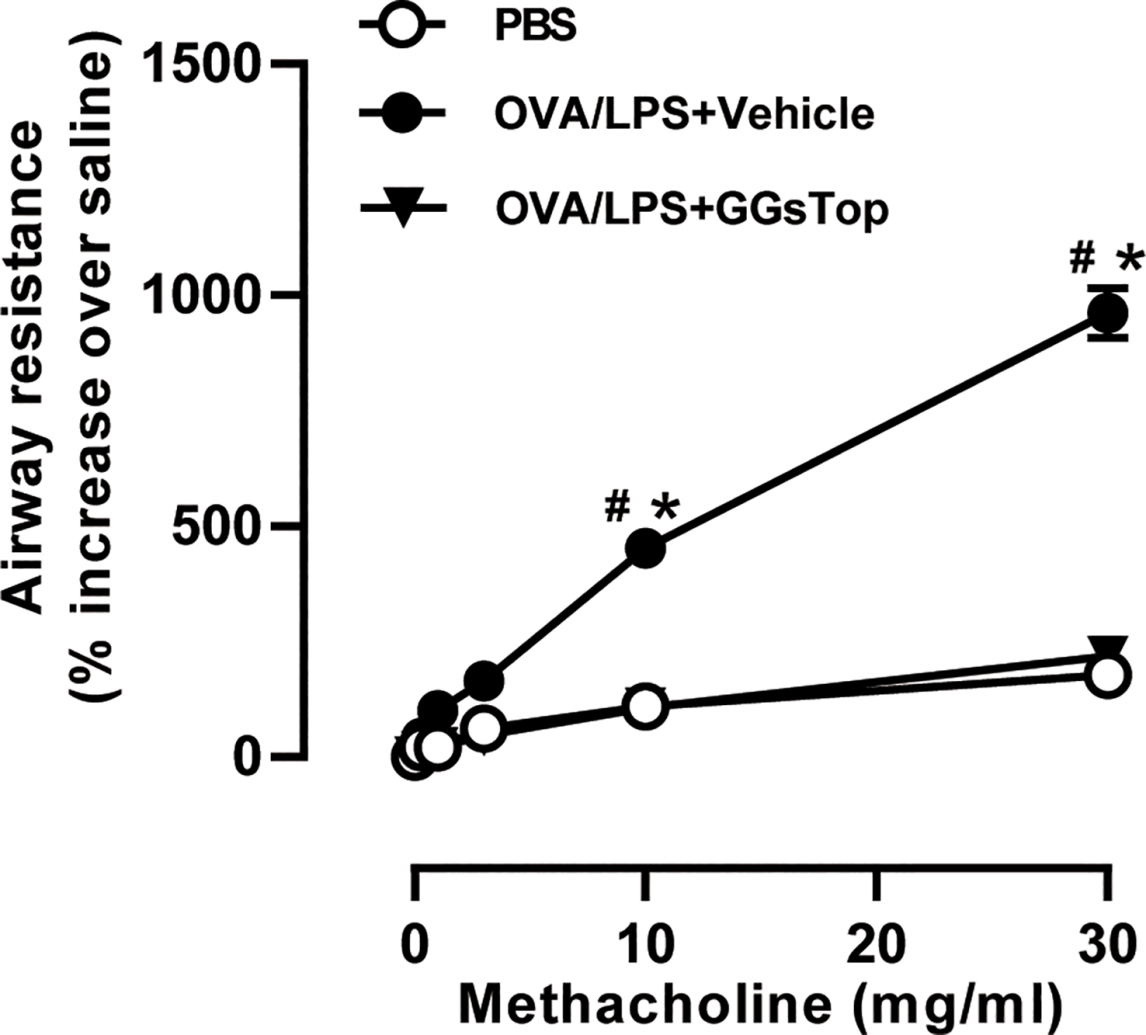
GGsTop treatment inhibits AHR in LPS-induced asthma exacerbation mice. Airway resistance was determined using flexivent platform, in response to incremental doses of nebulized methacholine represented as a percentage increase over baseline (PBS only) readings. Values are represented as mean ± SEM. PBS group had 4 mice, OVA/LPS/Vehicle had 5 mice; OVA/LPS/GGsTop had 5 mice. All experiments shown are representative of three independent experiments. ^#^Designates significant differences compared to both PBS and OVA/LPS/Vehicle groups (^#^p < 0.05). *Designates significant differences compared to the PBS group (*p < 0.05).

**Figure 2:**
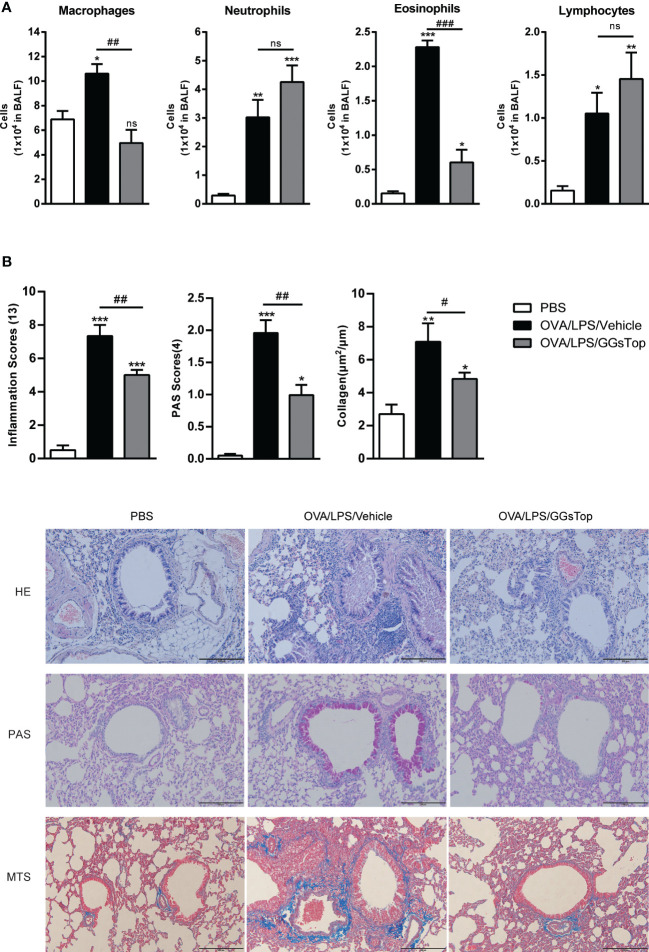
GGsTop treatment reduces inflammation, mucus hypersecretion, and collagen deposition in airways. **(A)** Differential BALF cell counts of neutrophils, macrophages, eosinophils, and lymphocytes presented as 1 × 10^4^ cells. **(B)** Representative images and scoring for HE, PAS, and MTS stained mouse lung sections. Values are represented as mean ± SEM. PBS group had 4 mice, OVA/LPS/Vehicle had 5 mice; OVA/LPS/GGsTop had 6 mice. All experiments shown are representative of three independent experiments. *Designates significant differences compared to PBS group (*p < 0.05, **p < 0.01, ***p < 0.001). ^#^Designate significant differences compared to paired group (^#^p < 0.05, ^##^p < 0.01, ^###^p < 0.001). ns represents non-significant differences.

Previous study has demonstrated that GGsTop attenuates IL-13-induced AHR and airway mucous production (20). To investigate the effects of GGsTop on airway inflammation and AHR, we employed our established mouse model of OVA and LPS induced steroid resistant asthma exacerbations (29). Mice were i.t. administered with GGsTop three days after LPS treatment in OVA/LPS/GGsTop group. To our surprise, GGsTop treatment abolished AHR in OVA/LPS group similarly to that in PBS group (**Figure 1**). Although GGsTop treatment had no effect on the infiltration of neutrophils and lymphocytes, it decreased the levels of macrophages and eosinophils in the BALF of OVA/LPS/GGsTop group (**Figure 2A**). Inflammatory scoring of lung tissue revealed an inhibitory role of GGsTop in promoting inflammation, as demonstrated by the markedly reduced inflammatory infiltrates in OVA/LPS/GGsTop group (**Figure 2B**). Furthermore, GGsTop administration drastically inhibited mucus hypersecretion and collagen deposition in airway (**Figure 2B**). The results suggest that GGsTop profoundly suppresses AHR, airway inflammation, and key features of tissue remodeling.

### GGsTop inhibits the expression of type 1 and 2 signature cytokines

**Figure 3:**
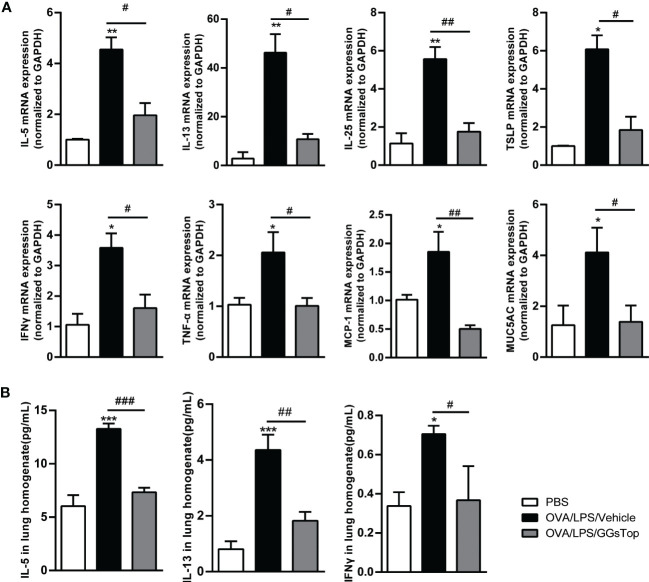
GGsTop suppresses the expression of proinflammatory factors. **(A)** mRNA expression levels of cytokines, MCP-1 and MUC5AC were assessed by qPCR and normalized to GAPDH. Presented as mean ± SEM. PBS group had 3 mice, OVA/LPS/Vehicle had 4 mice; OVA/LPS/GGsTop had 4 mice. **(B)** Protein levels of IL-5, IL-13 and IFNγ in lung tissues were assessed by CBA. Presented as mean ± SEM. PBS group had 4 mice, OVA/LPS/Vehicle had 4 mice; OVA/LPS/GGsTop had 4 mice. *Designates significant differences compared to PBS group (*p < 0.05, **p < 0.01, ***p < 0.001). ^#^Designate significant differences compared to paired group (^#^p < 0.05, ^##^p < 0.01, ^###^p < 0.001).

As type 1 and 2 cytokines underpin the development of asthma exacerbation (29), we investigated the effect of GGsTop on the expression of cytokines in lung by qPCR. OVA/LPS-treated mice exhibited increased significantly higher IL-5, IL-13, IL-25, TSLP and IFNγ mRNA in lung than those in PBS group (**Figure 3A**). Compared with OVA/LPS/vehicle group, GGsTop treatment was found to reduce the transcript levels of these five molecules. CBA assay confirmed the suppression of IL-5, IL-13 and IFNγ (**Figure 3B**). Interestingly, GGsTop similarly suppressed TNFα, MCP-1 and MUC5AC in the lung of OVA/LPS/GGsTop treated mice (**Figure 3A**). These results are indicative that GGsTop treatment reduces the expression of key inflammatory cytokines.

### GGsTop restores the level of glutathione

**Figure 4:**
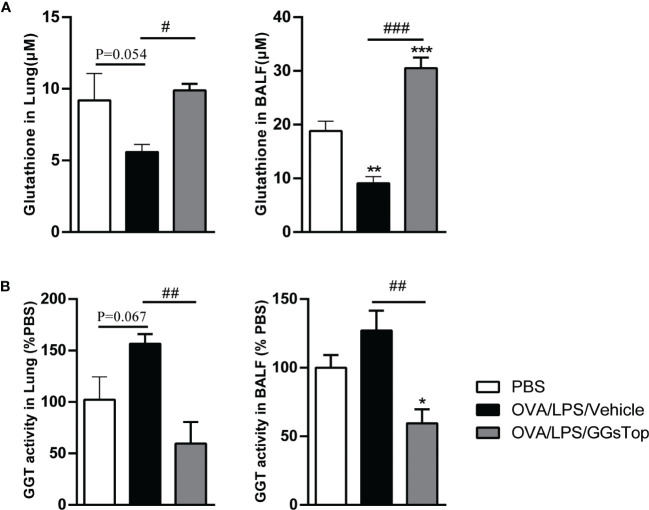
GGsTop restores glutathione generation in lung. The levels of glutathione **(A)** and GGT activity **(B)** were measured in lung and BALF. Data of GGT activity were expressed as percentage versus the control group. Presented as mean ± SEM. PBS group had 4 mice, OVA/LPS/Vehicle had 4 mice; OVA/LPS/GGsTop had 4 mice. *Designates significant differences compared to PBS group (*p < 0.05, **p < 0.01, ***p < 0.001). ^#^Designate significant differences compared to paired group (^#^p < 0.05, ^##^p < 0.01, ^###^p < 0.001).

As GGT is critical for the degradation of glutathione whose reduction is linked to asthma (38–40), we determined the levels of glutathione and GGT activity in both BALF and lung tissue. Although the differences of reduced glutathione and GGT activity in lung between OVA/LPS/Vehicle and PBS groups did not reach significance, the P values were close to 0.05. Nevertheless, OVA/LPS treated mice had significantly reduced level of glutathione in BALF (**Figure 4A**). GGsTop significantly suppressed the increased GGT activities in OVA/LPS/GGsTop treated mice in both BALF and lung tissue, as compared to those in OVA/LPS/Vehicle treated mice (**Figure 4B**). These results suggest that reduced levels of glutathione contribute to the pathogenesis and restoring glutathione generation by GGsTop could be beneficial for asthma treatment.

### GGsTop suppresses increase of eosinophils in peripheral blood and bone marrow

**Figure 5:**
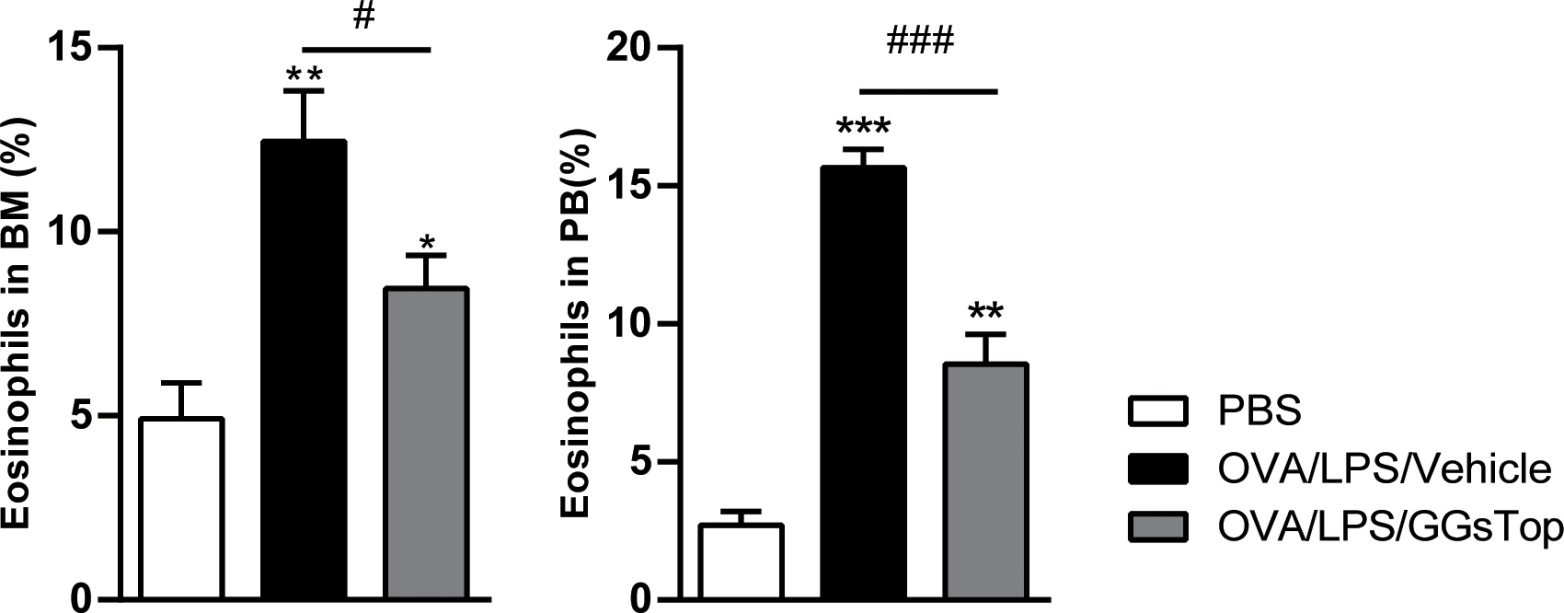
GGsTop reduces the increase of eosinophils in the bone marrow and peripheral blood. Differential percentages of eosinophils were calculated in the bone marrow (BM) and peripheral blood (PB). Presented as mean ± SEM. PBS group had 4 mice, OVA/LPS/Vehicle had 5 mice; OVA/LPS/GGsTop had 5 mice. *Designates significant differences compared to PBS group (*p < 0.05, **p < 0.01, ***p < 0.001). ^#^Designate significant differences compared to paired group (^#^p < 0.05, ^###^p < 0.001).

Eosinophils are notable immune cells involved in allergic asthma exacerbation and are markers for predicting exacerbation (41). We then assessed if GGsTop had any effect on eosinophil populations in bone marrow and peripheral blood where eosinophils mature and traffic (**Figure 5**). OVA/LPS/Vehicle treatment induced significantly elevated level of eosinophils in both compartments, as compared to that of PBS treatment. Although significantly higher than those of PBS treated mice, GGsTop greatly inhibited the increased levels of eosinophils in bone marrow and peripheral blood of OVA/LPS/GGsTop treated animals, suggesting that this inhibitor has systemic effect on eosinophilic inflammation during asthma exacerbations.

### GGsTop inhibits multiple immune factors and inflammatory pathways

**Figure 6:**
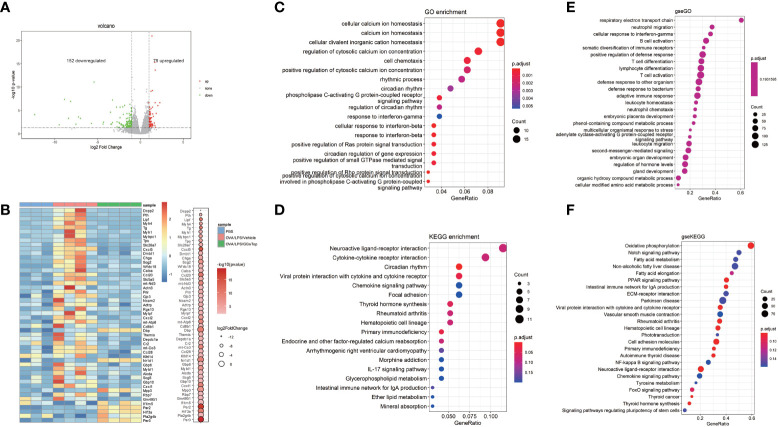
Gene-expression profiles influenced by GGsTop treatment. **(A)** The volcano plot shows 152 down-regulated genes and 79 up-regulated genes of OVA/LPS/GGsTop group compared with OVA/LPS/vehicle group. **(B)** The heat map depicts the top 50 significant DEGs in PBS group, OVA/LPS/vehicle group and OVA/LPS/GGsTop group. DEGs are differential expression in OVA/LPS/GGsTop group compared with OVA/LPS/vehicle group. **(C)** Gene ontology, **(D)** KEGG, and **(E, F)** GSEA analysis in OVA/LPS/GGsTop group compared with OVA/LPS/vehicle group based on the RNA sequencing data.

As GGsTop extensively inhibited hallmarks of asthma exacerbations, we employed RNA-seq to understand how GGsTop negatively regulated proinflammatory pathways and overcame steroid resistance induced by OVA/LPS, by examining RNA isolated from the bluntly dissected airway of treated mice. 152 down-regulated genes and 79 up-regulated genes were found to be differentially expressed of OVA/LPS/GGsTop group compared with OVA/LPS/vehicle group (**Figure 6A**). Of these genes identified, down-regulated factors included the immune factors CXCL1, CXCL2, and CXCL5, the macrophage inflammatory protein CCL20, and the pattern-recognition associated protein DMBT1 (42). Similarly, up-regulated factors included the interferon-related factor IFITM5, HIF3α, and Nr1d1 (**Figure 6B**).

Gene ontology (GO) and cell signaling pathway analysis (KEGG) revealed several differentially regulated pathways, most notably pathways associated with calcium ion homeostasis, cell chemotaxis, and cytokine-cytokine receptor interactions (**Figures 6C, D**). Calcium ion homeostasis has been proved to function in asthma. Ca2^+^ sensor STIM1 drives airway hyperresponsiveness and airway smooth muscle remodeling in asthma (43). Ca2^+^release–activated Ca2^+^ channel protein ORAI1 promotes Th2 cell functions in asthma (44). Cell chemotaxis and cytokine-cytokine receptor interactions are common knowledge that participate in inflammation response in asthma. Gene set enrichment analysis (GSEA) also enriched numerous pathways associated with Respiratory electron transport chain, oxidative phosphorylation (**Figures 6E, F**). Respiratory electron transport chain and oxidative phosphorylation are related to oxidative stress. Oxidative stress causes damage of lung promoting airway hyperresponsiveness and inflammation in asthma exacerbations. The molecular mechanisms have been studied extensively (45).

**Figure 7:**
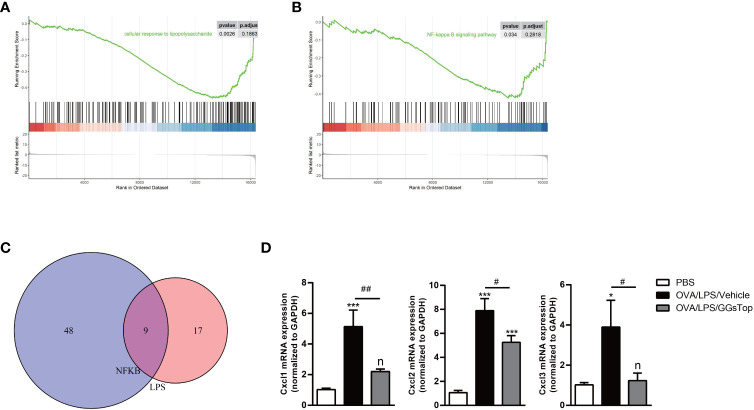
GGsTop treatment represses chemokines regulated by LPS/NFκB pathway. **(A, B)** GSEA analysis enriched cellular response to LPS and NFκB signaling pathway. **(C)** Venn plot showing the overlap of enriched genes between cellular response to LPS and NFκB signaling pathway. **(D)** Levels of CXCL1, CXCL2, and CXCL3 were assessed by qPCR and normalized to GAPDH. Presented as mean ± SEM. PBS group had 3 mice, OVA/LPS/Vehicle had 4 mice; OVA/LPS/GGsTop had 5 mice. *Designates significant differences compared to PBS group (*p < 0.05, ***p < 0.001). ^#^Designate significant differences compared to paired group (^#^p < 0.05, ^##^p < 0.01).

LPS binds to TLR4, and downstream activates transcriptional factors NFκB, IRF3, and JNK/AP1 (46, 47). GSEA analysis identified cellular response to LPS and NFκB signaling pathway was suppressed by GGsTop treatment (**Figures 7A, B**). To testify whether GGsTop functions by LPS/TLR4/NFκB pathway, the expression pattern of enriched genes involved in cellular response to LPS and NFκB signaling pathway gene sets in the analysis database were selected (**Figure 7C**; **Supplementary Figure S1**). Of the overlapping genes identified, the down-regulated chemokines CXCL1, CXCL2, and CXCL3 were further confirmed by qPCR (**Figure 7D**).

## Discussion

Here, we identified that GGsTop attenuated LPS-induced exacerbation on the basis of OVA-induced asthma, resulting in profound reductions of AHR, mucus hypersecretion, and the expression of multiple key proinflammatory factors. Our previous study has demonstrated that OVA/LPS induced AHR, and steroid-resistant airway inflammation (29). This demonstrates how GGsTop subverts asthma exacerbations in this treatment-resistant phenotype. Furthermore, our study demonstrated that GGsTop inhibits these hallmark features of airway inflammation, including heightened infiltration of macrophages and eosinophils in BALF, by down-regulating IFNγ and TLR4-related pathways that have previously been demonstrated to be hallmark factors involved in the perpetuation of steroid-resistant phenotypes (48).

Asthma is being increasingly recognized as a highly heterogeneous chronic disease with distinct etiology and diverse underlying mechanisms. GGT is a vital enzyme not only for metabolism and catabolism of glutathione but also for ROS production and leukotriene production in many diseases. Previous studies have demonstrated that GGsTop restored GSH levels and suppressed ROS production in ischemia/reperfusion-induced renal injury and neuroinflammation (21, 24). Indeed, increased level of GGT in serum is associated to oxidative stress and high levels of leukotrienes (49, 50), both of which are implicated in the pathogenesis of asthma (51, 52). Although there is no direct evidence linking GGsTop to leukotriene biosynthesis in asthma, we have shown that this inhibitor increases GSH level in the lung. GSH is closely associated with leukotriene production (53), implicating its’ role in asthma.

Similarly, the development of steroid resistance in asthma is heterogeneous. Potential mechanisms underlying steroid resistance are associated with multiple immune dysregulations. Type 2 cytokines (e.g., IL-4/IL-13) are found to be highly expressed in the lung of steroid resistant asthmatics (54–56). Type 1 cytokine, IFNγ inhibits GCR nuclear translocation, resulting in steroid resistance (27). IFNγ and TNFα induce steroid resistance in human airway smooth muscle cells *via* NFκB and STAT1 pathways (57). Dysregulated IL-10 production in lymphocytes and reduced HDAC activity in macrophage are similarly associated with steroid resistance in the pathogenesis (58, 59). Asthmatics resistant to steroid therapy demonstrate high levels of Th17 cells and IL-17A, which in turn regulate neutrophilic inflammation in airway. Furthermore, neutrophilic asthmatics often experience bacterial infection (60, 61). Interestingly, staphylococcal endotoxins B that is one of bacterial produces may induce steroid resistance by increasing the expression of GCR-β (62).

This mouse model of OVA/LPS-induced asthma exacerbation is characterized by heterogeneous inflammation, notably concerning the elevation of type 1 and type 2 cytokines which lead to AHR and steroid resistance (29). The common-known mechanisms of exacerbated asthma are mediated by induction of type 1 responses and/or type 17 responses, potentiation of type 2 responses, and elevated production of complex inflammation cytokines (29). TSLP, IL-5, IL-13 and IL-25 are the major proinflammatory cytokines that mediate eosinophilia-dominated type-2 inflammation (63). IL-5 can promote the proliferation, activation, and survival of eosinophils, while IL-13 administration to the lung can induce eosinophil recruitment, AHR, and mucus hypersecretion (64). IL-25 and TSLP are important epithelial cytokines to enhance downstream allergic lung inflammation by activating ILC2 cells (65). Like glucocorticoid treatments, GGsTop was able to reduce expressions of these type 2 cytokines in allergic lungs to mediate asthma attenuation. IFNγ expression is heightened in patients with a severe asthma phenotype (66), and administration of recombinant IFNγ to mice with induce AHR that is steroid-resistant (28). TNFα and MCP-1 expression is linked to neutrophilic asthma and is refractory to steroid treatment in severe/exacerbated patients (32, 67, 68). Both IL-1 and IL-6 are implicated in the pathogenesis of asthma. Although LPS administration alone increases the expression of IL-1a and IL-6 in the lung, the combination of OVA and LPS challenge reduces the expression of these two cytokines (69). Furthermore, we did not observe increased expression of IL-1a, IL-1b, and IL-6 in OVA/LPS treated mice (29). Different to glucocorticoid treatments, GGsTop was able to suppress expressions of IFNγ, TNFα and MCP-1, the three key factors in steroid resistant asthma. This demonstrates that γ-glutamyl transferase is implicated in asthma exacerbation through the regulation of a broader range of inflammatory chemokines and cytokines.

Interestingly, while we saw reductions in eosinophils and macrophages in the BALF, populations of neutrophils and lymphocytes were unchanged in response to GGsTop treatment despite improvements in inflammatory scores, AHR, mucus hypersecretion, and collagen deposition. This could be an indication that neutrophilic inflammation occurs independent of TLR4 activation, albeit with reduced pathogenicity. However, further studies are needed to investigate the dissociation between neutrophilic inflammation and other pathological changes in lung during asthma exacerbations. Surprisingly, treatment with GGsTop reduced eosinophil proportions in peripheral blood and bone marrow, even though it was directly administered into the lungs. This suggests that GGsTop not only suppresses Th2 responses in lung, but also has a systemic impact. Further studies are needed to elucidate whether its’ effect on the levels of eosinophils in the peripheral and bone marrow is direct or indirect. We have performed the assessment and found that GGsTop inhibits GGT activity in BALF, and reduced glutathione are increased in BALF after GGsTop treatment. Numerous studies have demonstrated the protective role of glutathione in lung diseases, including asthma, smoke-induced inflammation, and bleomycin-induced lung fibrosis (39, 40, 70, 71). These investigations support our finding that GGsTop improve asthma outcomes potentially *via* restoring glutathione level.

To understand the transcription mechanisms that are affected by GGsTop, treated mouse airways were collected and RNA-sequencing was performed to elucidate the presence of enriched pathways. Airways but not whole lung tissue were chosen to better understand airway inflammation, as they are the primary sites of the disease. LPS inhalation is one of important hazards for asthma exacerbation by stimulating many cells in airway, including airway epithelial cells and macrophages (31, 72, 73). LPS alone evokes features of asthma in mice, including airway inflammation, AHR and tissue remodeling (74). LPS binds to its receptor TLR4, resulting in LY96/MD-2 and CD14 transduction which facilitates NFκB activation, cytokines secretion, and inflammatory response (46, 47). Previous studies have confirmed that NFκB signaling pathway is activated in response to inflammatory stimuli during viral or bacterial asthma exacerbations (75). Our RNA-seq results confirmed the activation of this pathway in OVA/LPS/Vehicle group. By using GSEA analysis, GGsTop treatment suppressed the activation of TLR4/NFκB pathway. Importantly, NFκB regulates a significant number of cytokines (IL-5, IL-13, TNFα), chemokines (CXCL1, CXCL2, CXCL3, CXCL5, CCL20, and MCP-1), mucins (MUC5AC), and enzymes (MMP9) (75). We have confirmed the expression of some of those proinflammatory molecules by qPCR, which were attenuated by GGsTop treatment.

In addition to NFκB signaling, GGsTop also inhibited multiple pathways that are critical in the asthma exacerbation (**Supplementary Figure S2**). For instance, notch signaling pathway is involved in asthma pathogenesis by regulating airway inflammation, AHR and mucus production (76, 77). This is demonstrated in a study of asthma exacerbation that a Notch inhibitor SAHM1 suppresses the disease (77). Additionally, Peroxisome proliferator-activated receptor (PPAR) signaling pathway up-regulates the expression of Muc5ac in airway epithelial cells and ST2 expression in Th2 cells, both of which critically contribute to asthma pathogenesis (78, 79).

Glucocorticoids remain as the mainstay treatment for asthma. Inhaled glucocorticoids bind to glucocorticoid receptor, leading to decreased proinflammatory factors and increased expression of anti-inflammatory mediators (80). However, there are currently no effective treatments for steroid-resistant asthma that is heterogenous and is underlined by multifactorial mechanisms. Surprisingly, GGsTop attenuated the LPS-induced asthma exacerbation, which cannot be controlled by dexamethasone (29). This suggests that GGsTop inhibits asthma independent of glucocorticoid pathway. Further analysis of the steroid resistance-associated genes in the GGsTop treated lung revealed that 46 of them were down-regulated (**Supplementary Figure S3A**). GO enrichment analysis showed that the 46 genes are involved in biological processes including apoptosis, gliogenesis and smooth muscle contraction, which are important for the pathogenesis (**Supplementary Figure S3B**). Apoptosis of inflammatory cells is a mechanism by which glucocorticoids resolve eosinophilic and T-cell mediated inflammation in asthma (81, 82). Angiogenesis is a prominent feature of the airway remodeling that occurs in asthmatic airways, in which the expansion of blood vessel formation, accompanied by fibrosis, leads to further chronic inflammation (83). Abnormalities in airway smooth muscle contraction underpin hypercontractility and airway remodeling in asthma (84, 85). Indeed, previous study has indicated that eosinophils isolated from steroid-resistant asthmatic patients are resistant to steroid-induced apoptosis (86). This further underscores the therapeutic potential of GGsTop in steroid-resistant asthma treatment.

In conclusion, we have described that GGsTop inhibits the hallmarks of asthma exacerbation, including AHR, eosinophilic inflammation, mucus hypersecretion and collagen deposition. GGsTop deactivates several proinflammatory pathways and restrains the production of type 1 and 2 cytokines. GGsTop inhibits these pathological changes and suppresses the expression of steroid resistance-associated molecules independent of glucocorticoid pathway. Our data suggest that GGsTop treatment may be an alternative and effective therapeutic approach for treating asthma exacerbation.

## Data availability statement

The datasets presented in this study can be found in online repositories. The names of the repository/repositories and accession number(s) can be found below: GSE217834 (GEO).

## Ethics statement

All experimental protocols were approved by the Animal Ethics Committee of Zhengzhou University (#ZZURIB20180120).

## Author contributions

CZ, HX, LH, LZ, GZ, FL and MY designed the research. CZ, HX, KN, LS, YYM, ZM, YM, YD and CW performed the research. CZ, HX, FL and YM analyzed data. CZ, HX, FL and MY wrote the paper. All authors contributed to the article and approved the submitted version.

## Supplementary material

Supplementary Figure 1 The expression pattern of enriched genes involved in cellular response to LPS (A) and NFκB signaling pathway (B) gene sets in GSEA analysis database.

Supplementary Figure 2 GSEA analysis enriched Notch signaling pathway and PPAR signaling pathway.

Supplementary Figure 3 Bioinformatics analyses of overlapping DEGs and steroid-resistant genes. (A) Venn plot and Heat Map showing the overlap of genes between DEGs and steroid-resistant (SR) genes. (B) GO analysis of the overlapping genes.
