# Supplementary figures and images for: Inhibition of γ-glutamyl transferase suppresses airway hyperresponsiveness and airway inflammation in a mouse model of steroid resistant asthma exacerbation
Source: Front Immunol. 2023 Jun 12;14:1132939. doi: 10.3389/fimmu.2023.1132939 (PMC10292800; doi:10.3389/fimmu.2023.1132939)

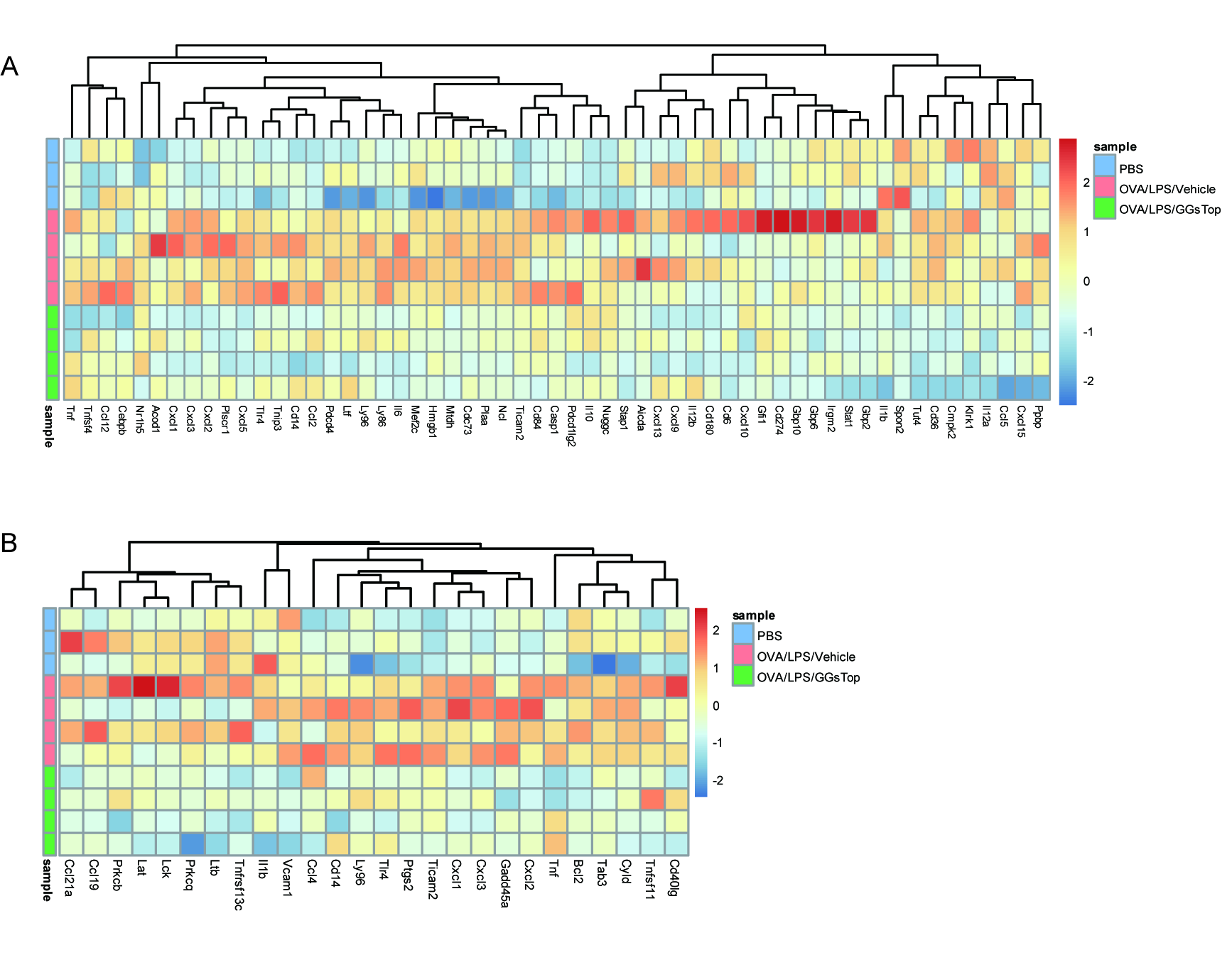

Supplement: Supplementary Figure 1 — The expression pattern of enriched genes involved in cellular response to LPS (A) and NFκB signaling pathway (B) gene sets in GSEA analysis database. [file Image_1.tif]

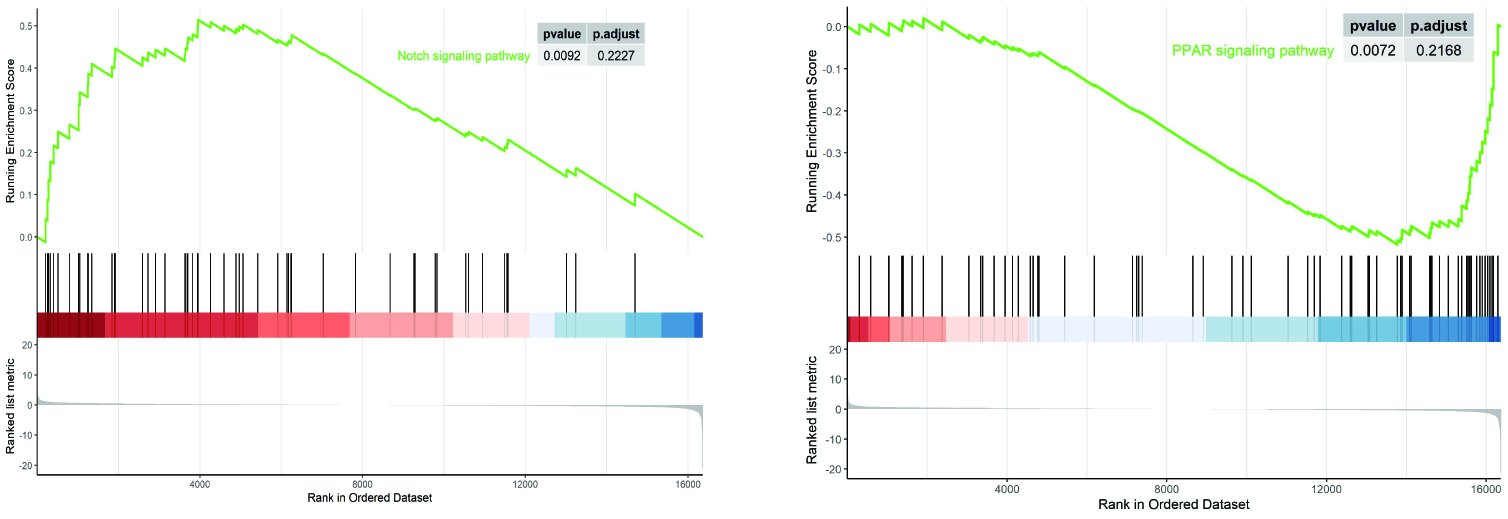

Supplement: Supplementary Figure 2 — GSEA analysis enriched Notch signaling pathway and PPAR signaling pathway. [file Image_2.tif]

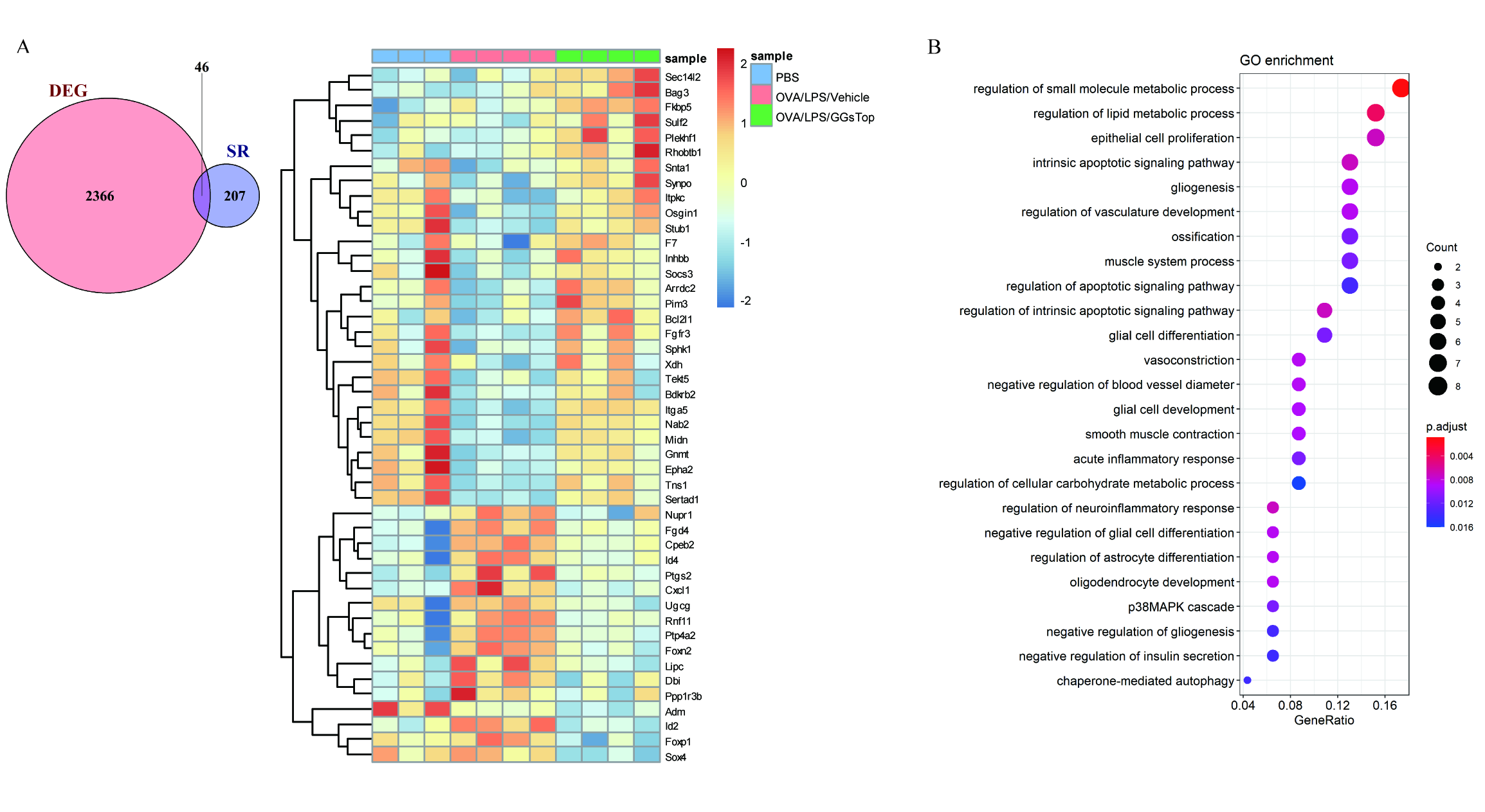

Supplement: Supplementary Figure 3 — Bioinformatics analyses of overlapping DEGs and steroid-resistant genes. (A) Venn plot and Heat Map showing the overlap of genes between DEGs and steroid-resistant (SR) genes. (B) GO analysis of the overlapping genes. [file Image_3.tif]
