# Supplementary material for: Inhibition of γ-glutamyl transferase suppresses airway hyperresponsiveness and airway inflammation in a mouse model of steroid resistant asthma exacerbation
Source: Front Immunol. 2023 Jun 12;14:1132939. doi: 10.3389/fimmu.2023.1132939 (PMC10292800; doi:10.3389/fimmu.2023.1132939)
Supplement: Supplementary file 4 [file Table_1.docx]

**Supplementary Table 1: qPCR primer sequences.**

| SYMBOL | Sequence (5’-3’) |
| --- | --- |
| GAPDH  Muc5ac  IL-13  IL-5  IL-25  TSLP  IFNγ  MCP-1  TNF-α  Cxcl1  Cxcl2  Cxcl3 | F: 5′-GGTGAAGGTCGGTGTGAACG-3′  R: 5′-CTCGCTCCTGGAAGATGGTG-3′  F: 5′-CTGTGACATTATCCCATAAGCCC-3′  R: 5′-AAGGGGTATAGCTGGCCTGA -3′  F: 5′-AGCTGAGCAACATCACACAAGACC-3′  R: 5′-TGGGCTACTTCGATTTTGGTATCG-3′  F: 5′-CTCTGTTGACAAGCAATGAGACG-3′  R: 5′-TCTTCAGTATGTCTAGCCCCTG-3′  F: 5′-AGTGTCCGGCATGTACCAGG -3′  R: 5′-TCCGGGGGTTCTTGCTCTTT -3′  F: 5′-GCGACAGCATGGTTCTTCTC-3′  R: 5′-TGCTCGAACTTAGCCCCTTT-3′  F: 5′‐TCTTGAAAGACAATCAGGCCATCA‐3′  R: 5′‐GAAT CAGCAGCGACTCCTTTTCC‐3′  F: 5′‐AGAGCCAGACGGGAGGAAG‐3′  R: 5′‐CCAGCCTACTCATTGGGATC‐3′  F: 5′‐GTCTACTGAACTTCGGGGTGATCG‐3′  R: 5′‐AGCCTTGTCCCTTGAAGAGAACC‐3′  F: 5´-TCCAGAGCTTGAAGGTGTTGCC-3´  R: 5´-AACCAAGGGAGCTTCAGGGTCA-3´  F: 5´-CATCCAGAGCTTGAGTGTGACG-3´  R: 5´-GGCTTCAGGGTCAAGGCAAACT-3´  F: 5´-TGAGACCATCCAGAGCTTGACG-3´  R: 5´-CCTTGGGGGTTGAGGCAAACTT-3´ |
